# Detection of ochratoxin A in human breast milk in Jiroft city, south of Iran 

**DOI:** 10.29252/cmm.3.3.1

**Published:** 2017-09

**Authors:** Ali Kamali, Sareh Mehni, Mohadeseh Kamali, Mehdi Taheri Sarvtin

**Affiliations:** 1 Department of Infectious Diseases, School of Medicine, Jiroft University of Medical Sciences, Jiroft, Iran; 2 School of Nursing and Midwifery, Jiroft University of Medical Sciences, Jiroft, Iran; 3 Department of Internal Medicine, School of Medicine, Kerman University of Medical Sciences, Kerman, Iran; 4 Department of Medical Mycology and Parasitology, School of Medicine, Jiroft University of Medical Sciences, Jiroft, Iran

**Keywords:** Breastfeeding, Breast milk, Ochratoxin A

## Abstract

**Background and Purpose:**

Breastfeeding plays an important role in the growth and development of infants. However, breast milk may be contaminated with various mycotoxins. Ochratoxin A is one of the most important mycotoxins with nephrotoxic, carcinogenic, teratogenic, genotoxic, and immunotoxic properties. Thus, we carried out this study to determine the concentration of ochratoxin A in human breast milk in Jiroft, Kerman Province, south of Iran.

**Materials and Methods:**

Eighty-four human breast milk samples were collected from mothers visiting the number one clinic in Jiroft city. Enzyme-linked immunosorbent assay (ELISA) was used to detect ochratoxin A in the samples.

**Results:**

Ochratoxin A was found in all the tested samples at a concentration ranging from 0.11 to 7.34 ng/ml. The mean concentration of ochratoxin A in the samples was 1.99±1.34 ng/ml**.** Fourteen samples contained ochratoxin A at concentrations exceeding the quantitation limit (3 ng/ml).

**Conclusion:**

The results of this study showed that infants are exposed to ochratoxin A in our region. In cases exceeding the quantitation limit, the infant's body cannot detoxify the toxin. Therefore, the infant can be affected by various illnesses such as nephropathy, immune system deficiency, and different types of cancer.

## Introduction

**Figure 1:**
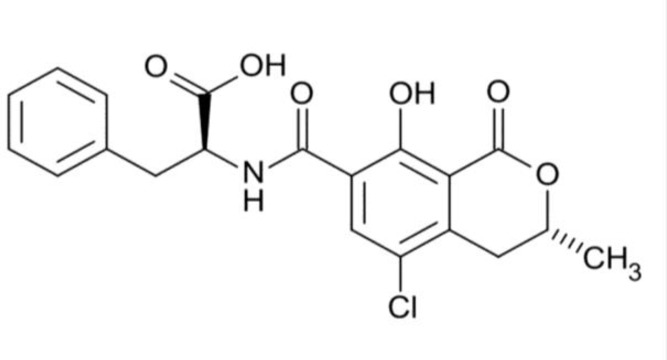
Chemical structure of ochratoxin A

Mycotoxins are toxic low-molecular-weight secondary metabolites produced by some fungal species [1, 2]. These metabolites constitute various groups of chemical compounds that can cause acute, subacute, or chronic diseases in humans [3]. So far, many studies have been performed on fungal toxins such as aflatoxins, ochratoxins, patulin, fumonisins, zearalenone, trichothecenes, and ergot alkaloids in foods, animals, and humans [3-9]. From myriads of mycotoxins, ochratoxin A (Figure 1) is one of the most important and deleterious mycotoxins produced by mold fungi, particularly *Aspergillus ochraceus, A. sulphureus, A. niger, Penicillium verrucosum, *and* P. nordicum* [10]*.* Ochratoxin A is a nephrotoxic, immunotoxic, hepatotoxic, and teratogenic mycotoxin [11]. The kidney is the major target organ in all the mentioned diseases [12]. Nephropathy and cancer are the most important effects of the toxin [13]. Ochratoxin A is mainly a nephrotoxic mycotoxin that is considered as the main etiological factor in Balkan endemic nephropathy, a lethal kidney disease related to the end stage of urothelial tumors [14]. This toxin is classified by International Agency for Research on Cancer as possible human carcinogen (group 2B) [15]. Ochratoxin A can cause cancer by the induction of oxidative DNA lesions coupled with direct DNA adducts through quinone formation [16]. In addition, ochratoxin A in human breast milk can elevate the risk of HIV acquisition in infants via the increase of mucosal HIV target cells and C-X-C motif chemokine 10 (CXCL10) concentration, as well as reduction in tumor necrosis factor-alpha (TNF-alpha) [17]. This toxin can be found in a variety of food commodities, including cereals, raisins, coffee, cocoa, wine, beer, fruits, and nuts [10, 11, 18, 19].

In the study by Tafuri et al. [18], ochratoxin A was recovered from 50% of cocoa powder samples at a concentration ranging from 0.22 to 0.77 μg/kg with a mean of 0.43 μg/kg. In Petkova‐Bocharova et al. study, ochratoxin A was recovered from 16.7% (25–27 μg/kg) of bean samples, 27.3% (25–35 μg/kg) of maize samples, and 9.0% (10–25 μg/kg) of wheat flour samples [19]. Ochratoxin A can be excreted from the body to human milk [20]. Breast milk is the most important food for infants [21]. Infants are considered to be more susceptible than adults to the effects of mycotoxins due to their higher metabolic rate, lower body weight, lower ability to detoxify, and incomplete development of some tissues and organs [11]. 

Jiroft city in southern Iran, which has a hot and humid climate, is suitable for growth of ochratoxin A-producing fungi. Currently, there is a scarcity of studies performed in Jiroft reporting the level of ochratoxin A in human breast milk. Therefore, we designed this study to investigate the level of ochratoxin A in human breast milk in the city of Jiroft, Kerman Province, south of Iran.

## Materials and Methods

In the present study, 84 human breast milk samples were collected from April 2016 to January 2017. Nursing mothers were selected from among the referrals to the number one clinic in Jiroft city, Kerman Province (Jiroft city is located in south-east of Iran). This city is 250 kilometers far from the province capital, and it has a warm and humid weather throughout the year that leads to the growth of various fungi. 

The inclusion criteria were healthy mothers with intention to breastfeed their infant. Mastitis and breast abscess were the exclusion criteria. All the mothers were informed of the study objectives and procedure and provided informed consent. About 5 cc of breast milk was taken from each subject. The samples were stored in sterile plastic containers at 4°C and then delivered to the laboratory in refrigerated boxes and frozen at −20°C until analysis. Before starting the experiments, the samples were thawed at laboratory temperature. Ochratoxin A was measured with enzyme-linked immunosorbent assay (ELISA) test kits (Europroxima, the Netherlands). According to the manufacturer’s instructions, breast milk samples were diluted 1:4 in dilution buffer, and 50 μl of the standard positive control and the diluted samples was dispensed into appropriate microplate wells. Then, 25 μl of conjugate (ochratoxin A-HRP) was added to each well. Afterwards, 25 μl of antibody solution was added to each well and incubated for 60 min in the dark at 37°C. 

The microplate wells were washed three times with rinsing buffer, and 100 μl of substrate solution was added to each well and incubated for 30 min at room temperature. The reaction was stopped by addition of 100 μl of stop solution. The absorbance of each well was measured at 450 nm. After that, the calibration curve was drawn and used to determine the concentration of ochratoxin A in each sample. According to the kit brochure, quantitation limit was less than 3 ng/ml. Results were analyzed in SPSS using descriptive statistics, Mann-Whitney U test, Kruskal-Wallis test, and Spearman correlation coefficient. *P-value* less than 0.05 was considered statistically significant.

The Research Ethics Committee of Jiroft University of Medical Sciences approved the study with ethical code of IR.jmu.REC.1394-22.

## Results

In this study, 84 human breast milk samples were examined. The mean ages of the mothers and infants were 28.88 years and 7.64 months, respectively. In this study, 50% of the infants were male, and 77 (91.7%) participants lived in urban areas. Forty-one (48.8%) infants were fed breast milk alone and 43 (51.2%) infants received breast milk and supplements. Thirty-two (38%), 38 (45.3%), and 14 (16.7%) mothers had under diploma, diploma, and academic degrees, respectively. Ochratoxin A was found in all (100%) the human breast milk samples (concentration range: 0.11-7.34 ng/ml). The mean concentration of ochratoxin A in the samples was 1.99±1.34 ng/ml. The concentrations of ochratoxin A in the human breast milk samples are shown in Table 1. 

The results of Kruskal-Wallis nonparametric test showed no significant association between the mothers’ level of education and ochratoxin A concentration (*P=0.69*). The mean concentrations of ochratoxin A recovered from samples of the city dwellers and suburban residents were 1.96 ng/ml and 2.27ng/ml, respectively. The results of Mann-Whitney U nonparametric test showed no significant difference between the city dwellers and suburban residents in ochratoxin A concentration (*P=0.244*).

**Table 1:** Concentration of ochratoxin A in human breast milk samples (ng/ml)

		**Number and percent of samples with ochratoxin A in ng/ml ranges**						**Exceeding EC/Codex regulation (** **3** ** ng/ml)**	
Samples tested (n)	Level of positive samples (%)	<1	1-2	2-3	3-4	4-5	>5	Number	Range (ng/ml)
84	84(100%)	17(20.2%)	36(43%)	17(20.2%)	7(8.3%)	2(2.4%)	5(5.9%)	14(16.7%)	3.06-7.34

Fourteen (16.57%) samples contained ochratoxin A at concentrations exceeding the quantitation limit (3 ng/ml). Samples of 12 (14.3%) city dwellers and 2 (28.6%) suburban residents had ochratoxin A at concentrations exceeding the quantitation limit. In this study, the subjects were aged 18 to 41 years old. The Spearman correlation coefficient showed a significant negative relationship between ochratoxin A concentration and maternal age (r=-0.3, *P=0.005*).

## Discussion

Although breastfeeding is highly important for normal growth and health of infants, breast milk can be contaminated with mycotoxins such as ochratoxin A that can cause serious diseases in infants [20]. Various studies from different regions revealed that human milk may contain different concentrations of ochratoxin A [11, 20, 22-25]. 

In this study, we investigated the concentration of ochratoxin A in human breast milk in Jiroft city. We detected ochratoxin A in all (100%) human breast milk samples. Jiroft city has a warm and humid climate with abundant precipitation, which coupled with varied plants and trees, makes it a suitable environment for many toxin-producing fungi. Fourteen samples containing higher amounts of ochratoxin A than the quantitation limit (3 ng/ml). 

In the study by Dehghan et al. [11], ochratoxin A was found in 84 (96.6%) samples, and 16% of the samples had higher amounts of ochratoxin A than the EU limits. In Dostal et al. [20] study, ochratoxin A was found in 23 (30.26%) samples. Nine (11.8%) positive samples were found to contain amounts higher than the quantitation limit. Our results were incompatible with those of Dostal et al. [20], but consistent with the findings of Dehghan et al. [11]. Moreover, in this study, the mean concentration of the toxin was higher than those in the previous studies, such as 0.0175 ng/ml in Brazil [22], 00.887 ng/ml in Egypt [23], 0.106 ng/ml in Chile [24], and 0.039.8 ng/ml in Norway [25], while the mean concentration of ochratoxin A in our study was lower than that in the study performed by Jonsyn et al. in Sierra Leone (7.9 ng/ml) [26]. 

The differences in results of various studies might be attributed to the moisture and temperature conditions, the number and types of fungi in each region, food storage methods, dietary habits, and measurement method of the toxin. There is limited information regarding the association between the level of education and concentration of this toxin in mothers. For education level, we divided the samples into three main groups of under diploma, diploma, and higher than diploma (collegiate). There was no significant association between the mothers’ level of education and ochratoxin A concentration. This result is incompatible with those of Dehghan et al. [11] study. This difference might be due to the lack of awareness of educated mothers about the toxin or the contamination of foods prepared outside the home. 

In this study, 50% of the infants were male. Baby boys need breast milk more than girls [27], hence boys are more exposed to the toxin than girls. The results of this study showed no significant difference in ochratoxin A concentration between residents of urban and suburban regions of the city. This finding is compatible with those of Dehghan et al. [11] and Skaug [28]. This result may be due to high similarity in climate and dietary habits in urban and suburban areas. In this study, the mothers were aged 18 to 41 years old. A significant negative relationship was noted between ochratoxin A concentration and maternal age. We showed that ochratoxin A concentration decreases with increasing maternal age. This result may be due to changes in dietary habits of older mothers. Furthermore, older mothers may have more knowledge regarding the toxin than younger mothers. In this study, 48.8% of the infants were fed breast milk alone and 51.2% of the infants received breast milk and supplements. It is expected that the toxic effects of ochratoxin A to be lower for those fed with breast milk and supplements due to the lower consumption of contaminated breast milk.

## Conclusion

In conclusion, the results of this study showed that the fungi producing ochratoxin A are present in our region and can cause food contamination with the production of ochratoxin A. In addition, it was discovered that food preservation in our region is probably not performed using the preferred method that has led to the growth of the toxin-producing fungi in them. Therefore, future studies are recommended to identify the fungi present in our area, as well as studies are required to examine the presence of this toxin in various foods. Contaminated foods should be identified and their consumption should be avoided. The amount of the toxin in human breast milk should be measured, and in case of high milk contamination, lactation should be avoided. To prevent the entrance of ochratoxin A into the body of infants, lactating mothers are recommended to use fresh foods.
